# Ag-migration effects on the metastable phase in CaCu_3_Ti_4_O_12_ capacitors

**DOI:** 10.1038/s41598-018-19241-0

**Published:** 2018-01-23

**Authors:** Ji-Won Lee, Gun-Hyun Lee, Dong-Jin Shin, Jinhwan Kim, Soon-Jong Jeong, Jung-Hyuk Koh

**Affiliations:** 1 School of Electrical and Electronics Engineering, Chung-Ang University, 84 Heukseok-ro, dongjak-gu, Seoul, Korea; 2 Korea Electrotechnology Research Institute, Changwon, 51543 Korea

## Abstract

The silver migration effect into the metastable phase forms a micro-electric path, to enhance the relative dielectric permittivity of CaCu_3_Ti_4_O_12_ ceramics for electronic devices. Controlling the sintering time uniquely develops the metastable phase of as-sintered CaCu_3_Ti_4_O_12_ ceramics. A post-heating process that applies the migration of silver into the metastable phase increases the relative dielectric permittivity. At 1 kHz frequency, the relative dielectric permittivity at room temperature of the silver-migrated CaCu_3_Ti_4_O_12_ ceramics sintered for 2 h is 565.9 × 10^3^, almost 52 times higher than that of the as-sintered CaCu_3_Ti_4_O_12_ ceramics. The selected area electron diffraction (SAED) patterns of the large and small grains were similar, but differed from those of the metastable region, including the grain boundary of the as-sintered CaCu_3_Ti_4_O_12_ ceramics sintered for 2 h by TEM technique. This phenomenon suggests that enabling Ag-migration into the metastable phase develops a micro-electric path that improves the relative dielectric permittivity of CaCu_3_Ti_4_O_12_ ceramics.

## Introduction

Due to their outstanding electrical properties, various perovskite titanate materials have played key roles in functional electronic devices^1^. Among these materials, calcium copper titanate (CCTO; CaCu_3_Ti_4_O_12_) ceramics have attracted considerable attention in recent years, due to their high relative dielectric permittivity^1–3^. The crystal structure of CCTO ceramics was identified as a perovskite-type body-centered cubic (bcc) structure^4^. Although numerous theoretical studies and experimental measurements have attempted to determine the reason for the high relative dielectric permittivity of CCTO ceramics, the mechanism of CCTO ceramic has not yet been clearly explained. Various models have been proposed to explain the high relative dielectric properties. For example, some research has shown that the CCTO structure is composed of conducting or semiconducting grains separated by thin insulating grain boundaries, which phenomenon is widely assumed to be the structural mechanism that leads to the high relative dielectric permittivity^5,6^.

Meanwhile, various techniques can be applied to increase the relative dielectric permittivity in a heterogeneous material, such as the transformation of the microstructure, or the formation of an electrical path for a contact network. In the case of microstructural transformation, a metallic material is used to form a large number of micro capacitors with many conducting particles separated by thin dielectric layers^7–10^. In addition, some reports have described the formation of an electric path between an insulator and metallic composite, which related to the conductivity of composite materials^11^. However, the artificial formation of an electric path by applying the migration effect of metal material in the ceramic materials has not yet been reported. Therefore, we focused on the formation of a micro-electric path in ceramic structure, which can be related to colossal relative dielectric permittivity. The migration effect can be used to form the micro-electric path into CCTO, and a metastable phase region is therefore required, where the migration process can occur. The metastable phase poses a fundamental question that is relevant in all disciplines that relate to solid state. Metastable phases can be formed by rapid quenching technique, or by various processes^12–15^. It is generally believed that ceramics develop a metastable phase when they do not get enough energy for the sintering process. Various processing parameters, such as sintering temperature, sintering time, and applied pressure, can be used to create the required metastable phase during the process. During the sintering process, the sintering time can be the easiest variable among these parameters to control the applied energy to obtain the metastable phase. Our previous study observed a metastable phase in CCTO sintered at 1125 °C for 2 h^16^. Therefore, controlling the sintering time can achieve a metastable phase of CCTO ceramics, and the migration of metallic material into the metastable phase can then generate the micro-electric path. Usually, silver (Ag) and nickel (Ni) are the representative migration materials^17,18^.

In this research, Ag was selected as the electrode and material for migration. The Ag-migration effects of CCTO ceramics that have a metastable phase can increase their relative dielectric permittivity. We assumed that migrating the metallic component into the metastable phase in the ceramic component, forms the micro-electric path, and as a result, enhances the capacitance. However, no experimental studies have been carried out on the difference in behavior between the migration effect and the dielectric property of CCTO ceramics.

This study controlled the sintering time at the optimized sintering temperature to observe the various phase conditions. Measurement of the relative dielectric permittivity, energy dispersive spectroscopy (EDS) and current density–electric field *(J–E)* properties in the fully sintered phase and partially sintered phase enabled the Ag-migration effect in CCTO to be analyzed and characterized.

## Results and Discussion

**Figure 1:**
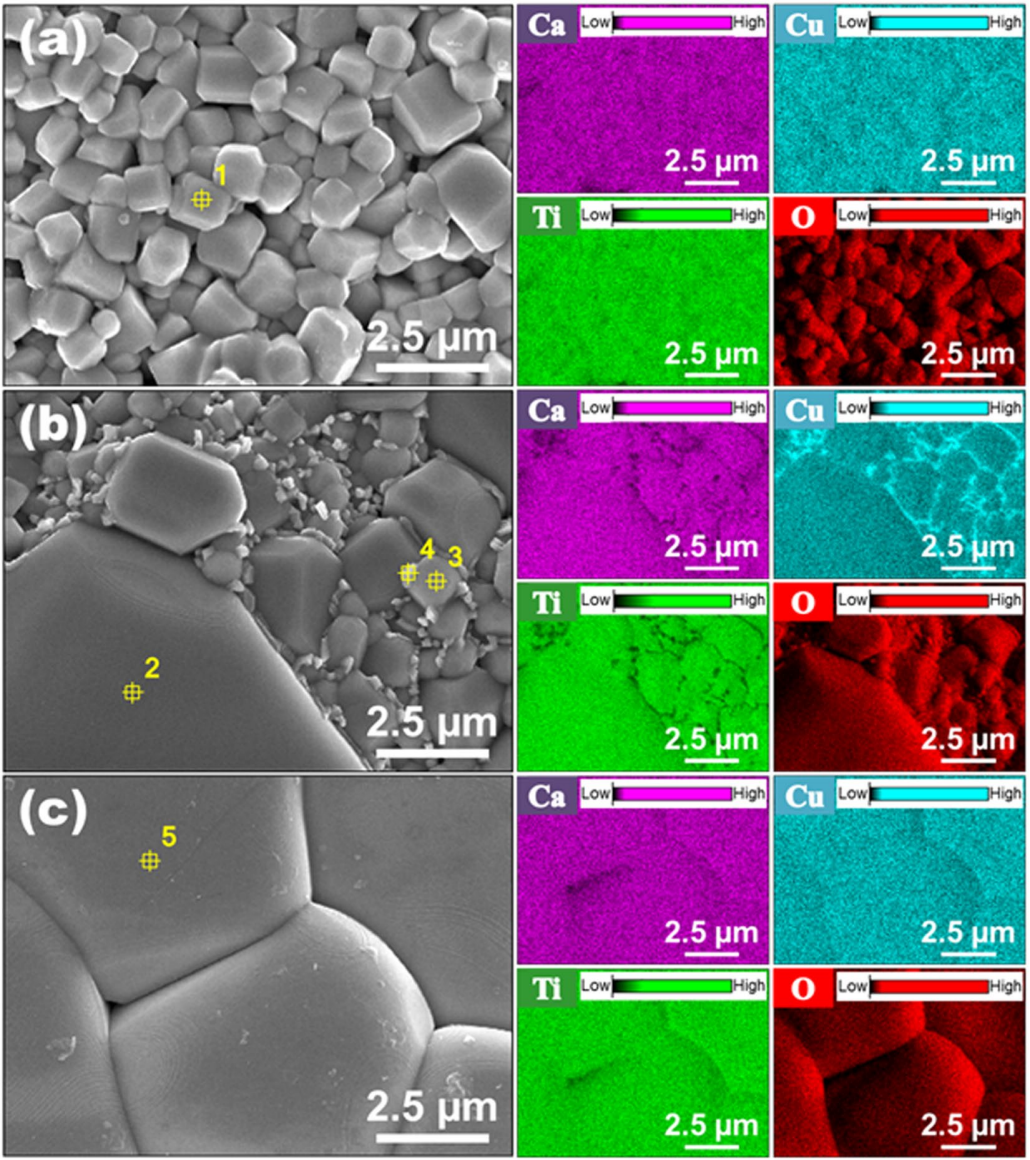
Surface morphology and chemical composition with various sintering times. Plane-view FE-SEM and EDS micrographs of the as-sintered CaCu_3_Ti_4_O_12_ ceramics sintered at 1125 °C in air for (**a**) 0.5 h, (**b**) 2 h, and (**c**) 12 h corresponding to the X-ray maps of calcium (Ca), copper (Cu), titanium (Ti), and oxygen (O).

We have attempted to control the phase of CCTO ceramics, expecting that controlling the sintering time could achieve a metastable phase of CCTO ceramics. The as-sintered CCTO ceramics were analyzed by FE-SEM and EDS to observe the surface morphology, determine the stoichiometric composition of small and large grains, and check the metastable phases. Figure 1a,b and c show the FE-SEM images for the surface microstructure of 0.5 h, 2 h, and 12 h sintered CCTO ceramics, respectively, without the post-heating process. Grain growth was not observed in the CCTO ceramics sintered for 0.5 h, except for the CCTO ceramics sintered for 2 h and 12 h. In addition, the average particle size of the 0.5 h sintered CCTO ceramics was around 1 µm. It was found that the increased sintering time could promote grain growth and dense microstructure^19^. While small grains were observed for the 0.5 h sintered CCTO ceramics, both small and large grains, as well as a metastable phase, were observed for the 2 h sintered CCTO ceramics, and very large grains were observed for the 12 h sintered CCTO ceramics.

The EDS analysis allows the stoichiometric composition in the various regions in the CCTO ceramics to be measured. Table 1 shows the measured stoichiometric composition of the as-sintered CCTO (Ca_1_Cu_3_Ti_4_O_12_) ceramics. The elemental contents of grain, grain boundary and metastable phase represented by error bars are shown in Figure S4. The measured composition ratio of small grains (Pt. 1, Fig. 1a) for the 0.5 h sintered CCTO almost matched the stoichiometric composition (Figure S1). However, increasing the sintering time to 2 h, resulted in some parts of the grains in the CCTO ceramics being grown as metastable phase. Figure 1b shows this phenomenon. The EDS result of the 2 h sintered CCTO ceramic showed the composition ratio in the large grain region (Pt. 2, Fig. 1b) to be similar to that of the small grain region (Pt. 3, Fig. 1b). These composition ratios in the large and small grains almost matched the stoichiometric composition. However, Pt. 4 in Fig. 1b showed a nonstoichiometric ratio, which is the metastable phase (Figure S2). The 12 h sintered CCTO ceramics showed relatively large grains, and no small grains, compared with those of other specimens. The EDS analysis showed that the large grains (Pt. 5, Fig. 1c) almost matched the stoichiometric composition (Figure S3). The FE-SEM and EDS results indicate that the longer sintering time contributes to grain growth and densification. Moreover, a metastable phase was not observed in the 0.5 h and 12 h sintered CCTO ceramics. The formation mechanism of the metastable phase can be explained as liquid phase sintering behavior which segregated Cu rich phase in case of CCTO ceramics. It was shown that Cu rich phase segregates out of CCTO grain towards the grain boundary and then forms metastable phase by increasing the dwell time. Cu rich phase was not segregated in 0.5 h sintered CCTO ceramic, but it was segregated to grain boundary region as the metastable phase with the sintering dwell time increased to 2 h. The segregated Cu rich phase was evaporated as the sintering time increases to 12 h.

**Table 1:** Chemical composition of grain, grain boundary and metastable phase for the as-sintered and Ag-migrated CaCu_3_Ti_4_O_12_ ceramics sintered at 1125 °C in air for 0.5 h, 2 h, and 12 h.

The as-sintered CCTO ceramics (Fig. 1)							
Element	Atomic percent						
	(a)	(b)				(c)	
	Grain	Grain	Grain	Metastable phase		Grain	
	Point 1	Point 2	Point 3	Point 4		Point 5	
Ca	5.49	5.77	5.90	2.56		5.98	
Cu	16.27	17.26	17.41	29.27		17.73	
Ti	23.36	24.84	25.04	10.70		25.50	
O	54.88	52.13	51.64	57.47		50.79	
**The Ag-migrated CCTO ceramics (Fig.** 3 **)**							
**Element**	**Atomic percent**						
	**(a)**		**(b)**			**(c)**	
	**Grain**	**Grain boundary**	**Grain**	**Metastable phase**	**Grain**	**Grain**	**Grain boundary**
	**Point 1**	**Point 2**	**Point 3**	**Point 4**	**Point 5**	**Point 6**	**Point 7**
Ca	4.62	4.51	5.93	4.84	4.30	6.53	6.88
Cu	15.56	10.23	17.11	23.25	12.72	19.92	16.89
Ti	20.84	18.25	24.43	19.90	18.47	28.08	31.12
O	58.98	66.82	52.53	51.66	64.51	45.47	44.99
**Ag**	**0**	**0.19**	**0**	**0.35**	0	**0**	**0.12**

**Figure 2:**
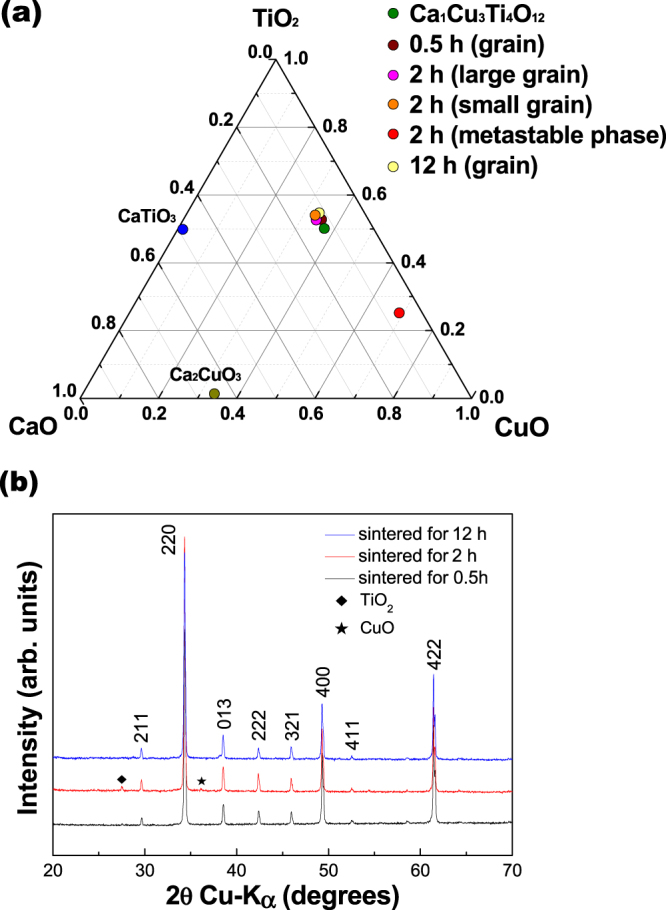
(**a**) Phase relations in the ternary system CaO-CuO-TiO_2_ for theoretical Ca_1_Cu_3_Ti_4_O_12_ ceramics, experimental 0.5 h, 2 h, and 12 h sintered CaCu_3_Ti_4_O_12_ ceramics depending on position. (**b**) X-ray diffraction profiles of the as-sintered CaCu_3_Ti_4_O_12_ ceramics sintered at 1125 °C in air for 0.5 h, 2 h, and 12 h.

In order to verify the distribution of the metastable phase, EDS elemental mapping was performed on CCTO ceramics sintered with different conditions. The EDS mapping shows a distinct distribution of specific elements within an inspection field, as indicated by unique colors. Also, the bright spots in the EDS mapping correspond to a high concentration of the mapped element. The EDS elemental mapping results show the degree of calcium (Ca), copper (Cu), titanium (Ti), and oxygen (O) concentration based on the SEM images in Fig. 1. The colors representing the Ca, Cu, Ti, and O elements are purple, cyan, green, and red, respectively. The Cu rich region, indicated as the brightest part of the EDS analysis, was only observed in the 2 h sintered CCTO ceramics, especially near the grain boundaries. The degree of brightness modulation corresponds to the intensity of stoichiometric composition in Table 1. We believe that this Cu rich region could form the metastable phase during the sintering process. In contrast to the 2 h sintered CCTO ceramics, no metastable phase region was observed in the CCTO ceramics sintered for 0.5 h and 12 h. The different grain size distribution and chemical composition of the CCTO specimens, as a result of the different sintering times, may have caused the differences in their dielectric and electrical properties after the Ag-migration process.

The ternary system CaO-CuO-TiO_2_ was reported for CCTO ceramics by Gibbs energy and XRD analysis^20,21^. Jacob *et al*. carried out the measurement of oxygen potential to determine the standard Gibbs energy of formation of CCTO. It was reported the presence of CaTiO_3_, Ca_3_Ti_2_O_7_, and Ca_4_Ti_3_O_10_ compounds along the CaO–TiO_2_ binary at 1000 °C in 0.165 (*P*o_2_/*P*°) ≤ 1. Also, Ca_2_CuO_3_ phase was present along the CaO–CuO binary. In order to explain the metastable phase of 2 h sintered CCTO, phase diagram was analyzed. Figure 2a shows the phase relations in the ternary system CaO-CuO-TiO_2_ for theoretical Ca_1_Cu_3_Ti_4_O_12_ ceramics and experimental 0.5 h, 2 h, and 12 h sintered CaCu_3_Ti_4_O_12_ ceramics depending on position. The phase diagrams are based on measured stoichiometric composition of the as-sintered CCTO in the Table 1. In Fig. 2a, the phases of all grains except for the metastable phase are close to theoretical Ca_1_Cu_3_Ti_4_O_12_ phase. It means that there is less possibility of the secondary phase existence in 0.5 h and 12 h sintered CCTO than 2 h sintered CCTO that has metastable phase.

Figure 2b shows the XRD patterns of the CCTO ceramics, which were sintered at 1125 °C for 0.5 h, 2 h, and 12 h. The XRD analysis shows that for the 0.5 h and 12 h sintered specimens, the CCTO ceramics have a perovskite structure without any pyrochlore phase. However in the 2 h sintered CCTO ceramics, secondary phases of TiO_2_ (27.5°) and CuO (36°) were observed. The secondary phases were reported such as CaTiO_3_, CuO, TiO_2_ depending on the sintering temperature and the mixing process by types of mixing medium in synthesizing^22,23^. In this study, TiO_2_ and CuO phases appear in the 2 h sintered CCTO ceramics, which can be attributed to the separated Cu phase.

**Figure 3:**
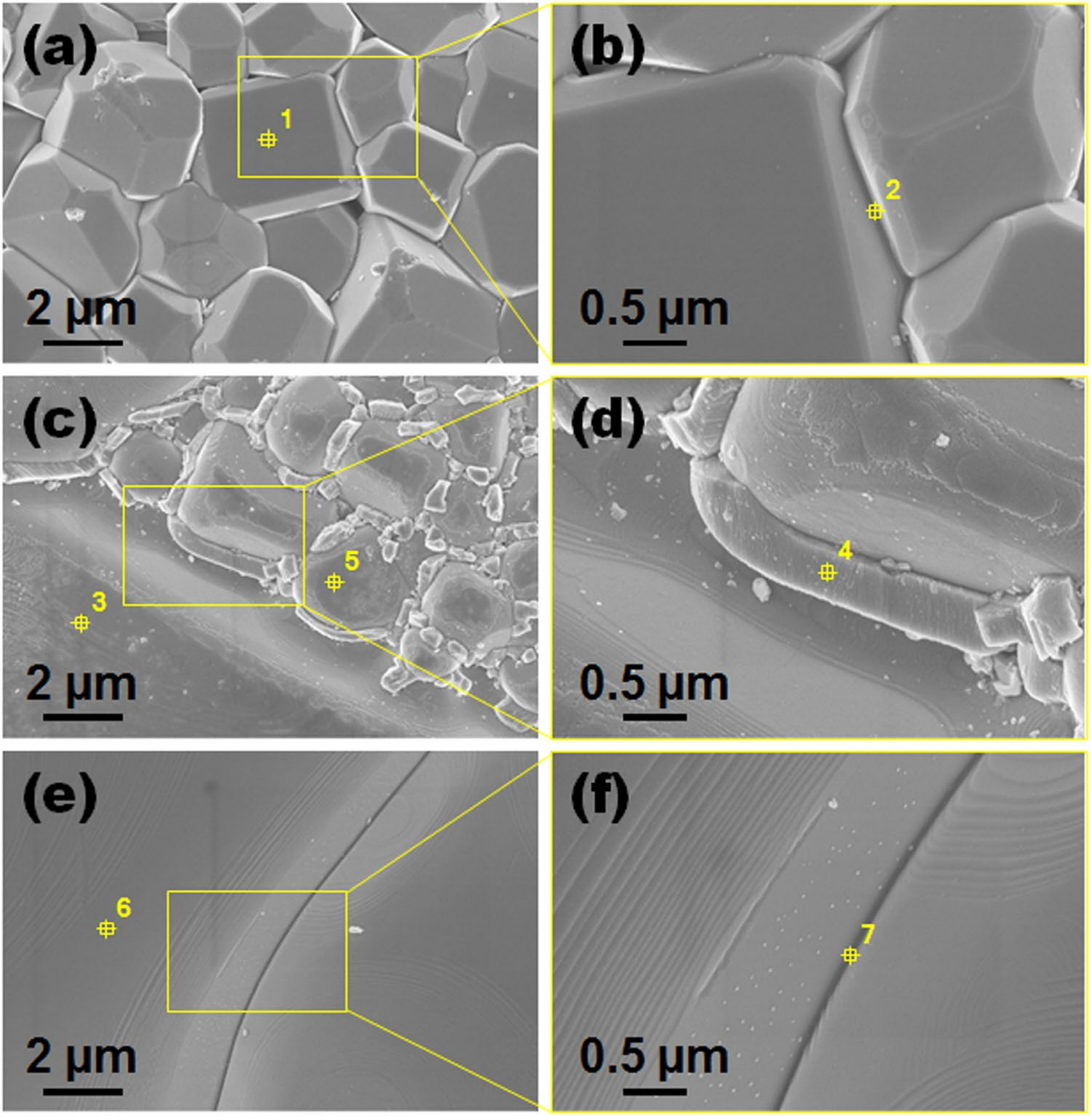
EDS analysis of FE-SEM images for the CaCu_3_Ti_4_O_12_ ceramics sintered at 1125 °C for (**a**) and (**b**) 0.5 h, (**c**) and (**d**) 2 h, and (**e**) and (**f**) 12 h after the Ag-migration process at 700 °C in air for 1 h.

In order to verify the Ag-migration effect on the metastable phase, EDS elemental mapping was performed on CCTO ceramics sintered with different conditions. Figure S8 shows the EDS micrograph of Ag-migrated CCTO ceramics. Although various attempts have been made to analyze the migrated Ag by EDS mapping, it has been analyzed only by the whole image. It is very difficult to identify the Ag on metastable phase and grain boundary using EDS mapping because the measurement limitation of EDS mapping and the amount of migrated Ag are quite small. Therefore, it is considered efficient to use chemical composition analysis and electrical characteristics. Figure 3a,c and e show the FE-SEM images for the Ag-migrated CCTO ceramics sintered at 0.5 h, 2 h, and 12 h, respectively. Figure 3b,d and f are magnified views of the Ag-migrated CCTO ceramics. Various points of the grains, grain boundaries, and metastable phase regions were checked (from Pts. 1 to 7), and their composition ratios were measured by EDS analysis. Table 1 shows the degrees of concentration of Ca, Cu, Ti, O, and Ag that were measured. Surprisingly, Fig. 3d shows the Ag element was detected in the metastable phase region including the grain boundaries (Pt. 4) in the 2 h sintered CCTO ceramics (Figure S6). Also, it was detected in the grain boundary region at Pt. 2 in Fig. 3b (Figure S5) and Pt. 7 in Fig. 3f (Figure S7). However, Ag element was not detected in the small and large grain region (Pts. 1, 3, 5 and 6). This means that in the 2 h sintered CCTO ceramics after the post-heating process, Ag element was migrated into the metastable phase. The migration can occur through the metastable phase or extended defects such as grain boundary. In general, the migration is more effective at the grain boundary rather than grain, because the activation energy of grain boundary is much smaller with the extended defects^24^. Thus, Ag can be expected to migrate through the metastable phase and grain boundaries compared to grain region where is chemically proportional.

**Figure 4:**
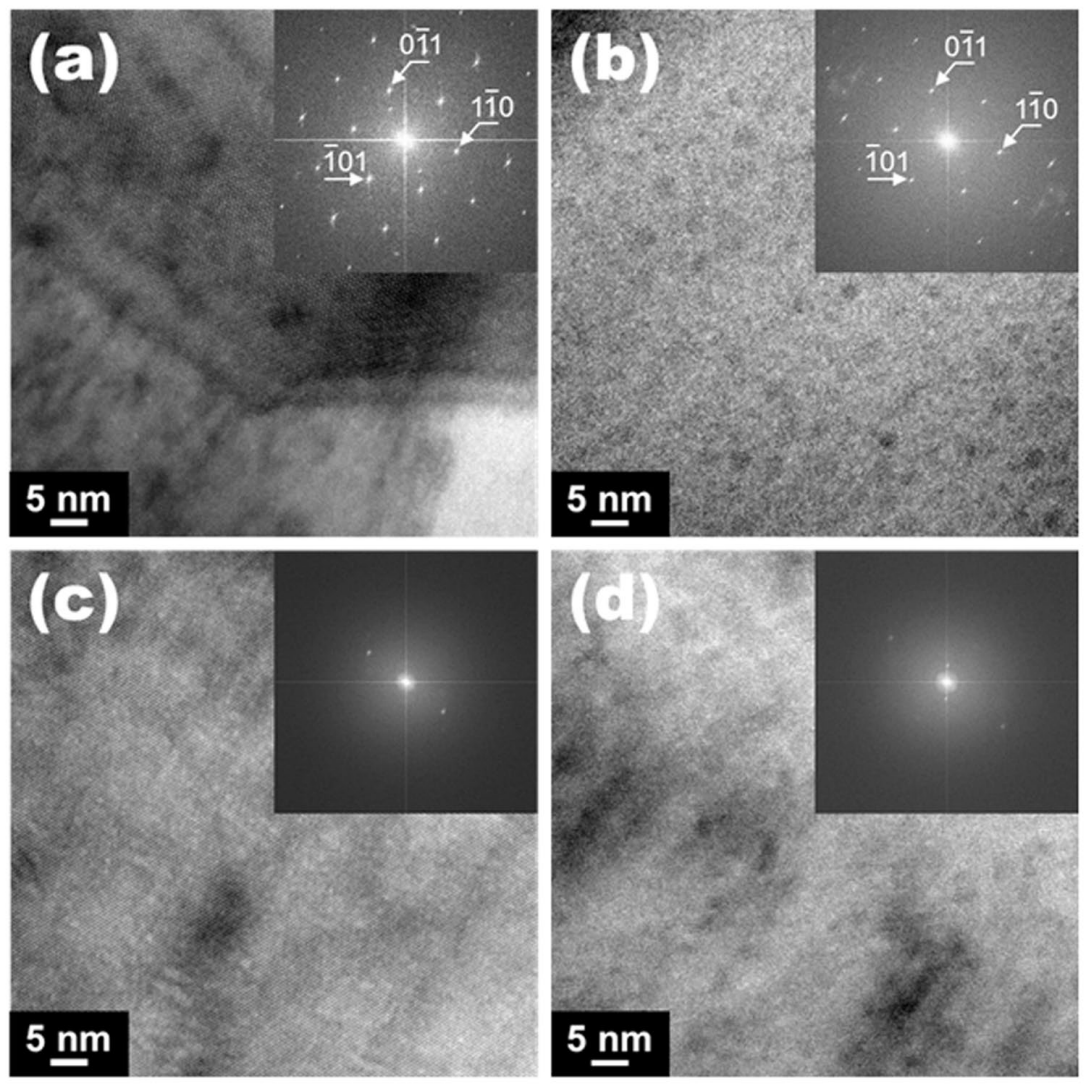
TEM images and selected area diffraction patterns of (**a**) large grain region, (**b**) small grain region, and (**c**) metastable phase region including grain boundary for the as-sintered CaCu_3_Ti_4_O_12_ ceramics sintered for 2 h. (**a**)–(**c**) correspond to Pts. 2–4, respectively, in Fig. 1b. TEM image and SAED pattern of (**d**) metastable phase region for Ag-migrated CaCu_3_Ti_4_O_12_ ceramics sintered for 2 h corresponding to Pt. 4 in Fig. 3d.

In order to confirm the existence of the metastable phase region and the migration effect, as-sintered and Ag-migrated CCTO ceramics sintered for 2 h were prepared for transmission electron microscopy (TEM). Figure 4a and b show the TEM images and the selected area electron diffraction (SAED) patterns of the large and small grains of as-sintered CCTO ceramics that correspond to Pts. 2 and 3 in Fig. 1b, respectively. The large and small grains show similar SAED patterns. Figure 4c shows the TEM image and SAED pattern of the metastable phase region of the as-sintered CCTO ceramics that corresponds to Pt. 4 in Fig. 1b. The SAED pattern of the metastable phase region differs significantly from those for the large and small grain sized CCTO ceramics. From the SAED pattern for Inserts a and b in Fig. 4, crystalline properties of (−101), (0–11) and (1–10) are evident. In contrast, crystalline properties are not evident, as shown in the SAED pattern of Insert c in Fig. 4. It seems that an appreciably different SAED spot appears, because the CCTO ceramic shows twinned and misoriented structure, and a nonstoichiometric composition dependent sintering process^25,26^. Moreover, there is contrast difference in Fig. 4c which defects are presented with increased contrast^27^. This means that the large and small grains have relatively crystalline properties, compared with those of metastable phase. EDS, XRD, and TEM analysis of the 2 h sintered CCTO ceramic clearly show a metastable phase region. In this metastable phase, the migration process can lead to the formation of the micro-electric path in the CCTO ceramic. Figure 4d shows the SAED pattern of the metastable phase region in the Ag-migrated CCTO ceramic, which corresponds to Pt. 4 in Fig. 3d. Although change of SAED patterns is apparent between the metastable phase regions of the as-sintered and Ag-migrated CCTO ceramics, we have not evidently detected the metastable phase. It is because of low dimensional Cu rich region and change the extinction of the reflections^28^. EDS analysis confirmed the presence of the migrated Ag in the CCTO ceramics.

**Figure 5:**
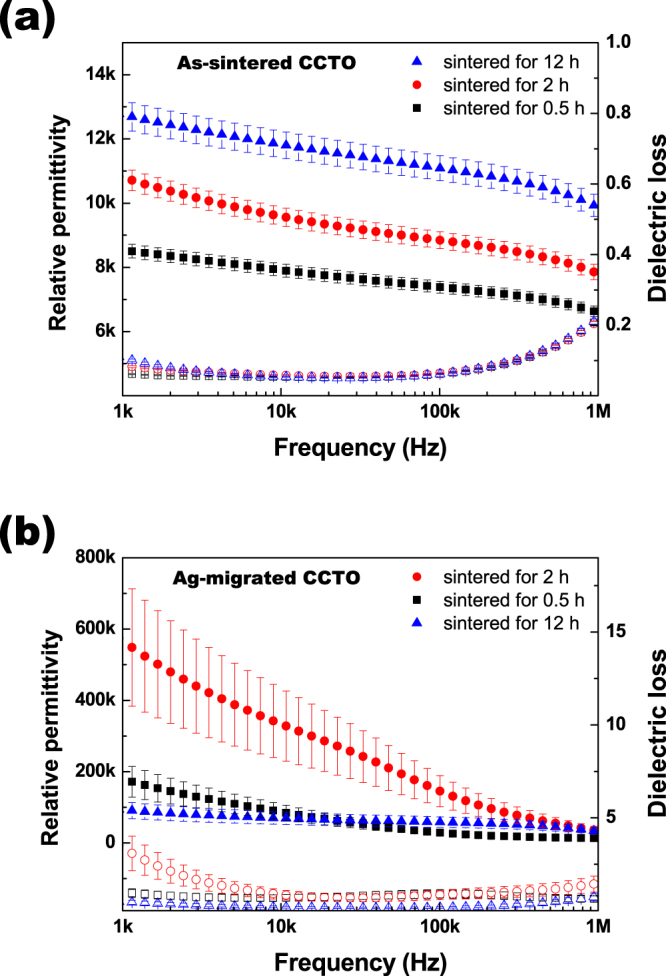
Frequency dependence of the relative dielectric permittivity and dielectric loss for the (**a**) as-sintered, and (**b**) Ag-migrated CaCu_3_Ti_4_O_12_ ceramics sintered at 1125 °C in air for 0.5 h, 2 h, and 12 h.

Figure 5a and b show the frequency dependences of the relative dielectric permittivity and the dielectric loss for the as-sintered and Ag-migrated CCTO ceramics, respectively, sintered at 1125 °C for 0.5 h, 2 h, and 12 h. Figure 5a shows that before the post-heating process, the relative dielectric permittivity ε_r_ at a frequency of 1 kHz of all the as-sintered CCTO ceramics was more than 8,500. The relative dielectric permittivity of the CCTO ceramics was increased with increasing sintering time. This could be due to the increased grain size, which is a result of the decreased porosity of the as-sintered CCTO specimens, as confirmed by the FE-SEM micrograph (Fig. 1). The relative dielectric permittivity for Ag-migrated CCTO ceramics was colossally increased after the post-heating process in air at 700 °C for 1 h, as seen in Fig. 5b. At a frequency of 1 kHz, the relative dielectric permittivities of Ag-migrated CCTO ceramics sintered for 0.5 h, 2 h, and 12 h were 178.3 × 10^3^, 565.9 × 10^3^, and 92.6 × 10^3^, respectively. In particular, the relative dielectric permittivity of Ag-migrated CCTO sintered for 2 h was almost 52 times that of the as-sintered CCTO ceramic; this Ag-migrated CCTO specimen has the highest value among the specimens. The metastable phase behavior was reported depending on sintering temperature and dwell time^29^. The relative dielectric permittivity of CCTO ceramic sintered at 1050 °C was higher than that of the CCTO ceramic sintered at 1100 °C. This is not in agreement with our study that the relative dielectric permittivity of the CCTO ceramics was increased with increasing sintering time, but may be associated with grain growth observed here. Moreover, it is a remarkable difference that the relative dielectric permittivity of CCTO ceramics has achieved a colossal growth employing Ag-migration effect on metastable phase and grain boundary by post-annealing process. Additionally, the increase of the relative dielectric permittivity of Ag-migrated CCTO sintered for 12 h was less than that of the Ag-migrated CCTO sintered for 0.5 h. Since the 0.5 h sintered CCTO ceramics have a greater number of grain boundaries than the 12 h sintered CCTO ceramics, Ag can more easily migrate into the grain boundaries. As a result, a much higher relative dielectric permittivity was observed, compared with the 12 h sintered CCTO. Figure S9 show the variation of the imaginary part (ε″) of the complex dielectric constant with frequency at different sintering time of the as-sintered and Ag-migrated CCTO ceramics.

**Figure 6:**
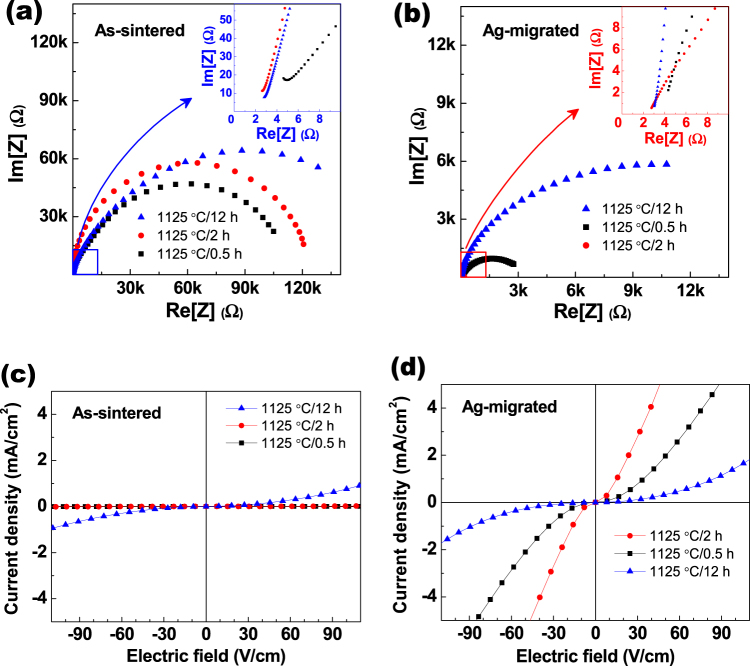
Cole-Cole plots of the (**a**) as-sintered, and (**b**) Ag-migrated CaCu_3_Ti_4_O_12_ ceramics sintered at 1125 °C in air for 0.5 h, 2 h, and 12 h. The insets show the magnified views of the high-frequency region close to the origin. Current density versus electric field of the (**c**) as-sintered, and (**d**) Ag-migrated CaCu_3_Ti_4_O_12_ ceramics.

In order to understand the impedance properties of the CCTO ceramics, the complex impedance measurements of as-sintered and Ag-migrated CCTO ceramics were analyzed. Figure 6a and b show the complex impedance spectra (Cole–Cole plots) of the as-sintered and Ag-migrated CCTO ceramics, respectively, sintered at 1125 °C for 0.5 h, 2 h, and 12 h. The Insets show magnified views of the high-frequency region close to the origin. Generally, impedance plots are composed of two semicircular arcs; the intercept of one arc on the real axis indicates the grain resistance (*R*_*G*_), while the intercept of the other arc indicates the grain boundary resistance (*R*_*GB*_)^30^. Frequency dependent impedance plots of the 0.5 h, 2 h, and 12 h sintered CCTO ceramics can be modeled as one very weak semicircle and one normal semicircle of the grain and grain boundaries, respectively. Therefore, the insets correspond to the impedance plots of high frequency, which are related to the grain properties. Figure 6a shows that the *R*_*GB*_ increased with increasing sintering time for the 0.5 h, 2 h, and 12 h as-sintered CCTO ceramics. Figure 6b shows that the Ag-migration process decreased the *R*_*GB*_ of all the CCTO specimens. In particular, the *R*_*GB*_ of Ag-migrated CCTO sintered for 2 h decreased from 120,000 Ω to 166 Ω; the measured resistance value was the lowest, and reduced to 0.138% of that without the post-heating process. We believe these drastically decreased resistance values probably derive from the Ag-migration effect into the CCTO specimen. The developed micro-electric path after the post-heating process helped to increase the capacitance values, as discussed, and as shown in Fig. 5. On the other hand, the without and with Ag-migration effect showed no significant difference in *R*_*G*_. The insets show the resistance of the grain region, which has no area for Ag-migration. Therefore, *R*_*G*_ in the grains region did not change with the introduction of the Ag-migration process. As a result, we are convinced that the Ag-migration phenomenon occurs into the metastable phase and the grain boundary area, rather than in the more stable phase, such as the grain area of CCTO ceramics.

Figure 6c and d show the electric field (*E*) dependent current density (*J*) plots of the as-sintered and Ag-migrated CCTO ceramics sintered at 1125 °C in air for 0.5 h, 2 h, and 12 h, respectively. The current density of the as-sintered CCTO ceramics increased with increasing sintering time. Before the post-heating process, the leakage current density of the 12 h sintered CCTO was higher than those of the other two specimens. In general, dielectric materials have a nonlinear current density (*J*) – electric field (*E*) relationship (*J–E*) that withstands leakage current to a certain level of threshold voltage. Therefore, by measuring the nonlinear *J*–*E* characteristic curve, the electrical properties of a specimen can be used to distinguish between the dielectric and conductor materials. The *J–E* properties of CCTO ceramics have been shown to have a strongly nonlinear relationship^4,31^. Figure 6d shows that the Ag-migration process colossally increased the leakage current density. Similarly, with the increase in relative dielectric permittivity, the 2 h sintered CCTO showed the highest current density among the three specimens, and the leakage current density reached almost 884 times higher than that of the as-sintered CCTO ceramic measured at 60 V/cm. Additionally, after the Ag-migration process the 0.5 h sintered CCTO ceramics showed a higher leakage current density than that of the 12 h sintered CCTO ceramics. These results indicate that the Ag-migration effect into the metastable phase, including the grain boundaries, colossally increased the current density. We believe this increased leakage current density probably derived from the developed micro-electric path into the metastable phase during the post-heating process.

**Figure 7:**
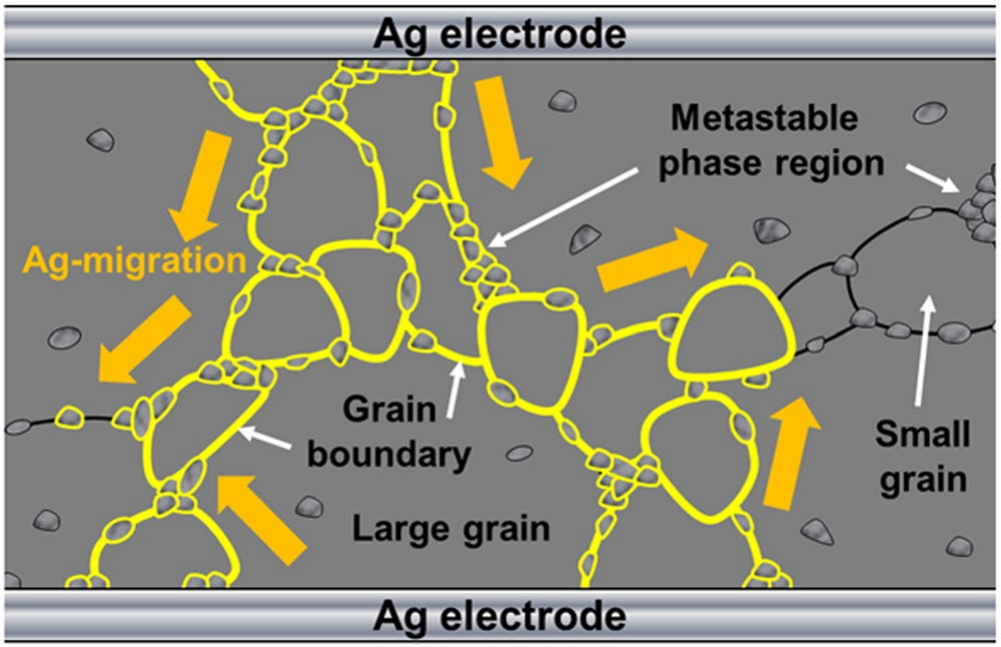
Schematic of the Ag-migration phenomenon into metastable phase including grain boundary for the 2 h sintered CaCu_3_Ti_4_O_12_ ceramics.

Figure 7 shows a schematic of the Ag-migration into the metastable phase region including a grain boundary for the 2 h sintered CCTO ceramics. The post-heating process can migrate Ag material into the metastable region, and form a micro-electric path through the metastable phase and grain boundary region.

We reported on the Ag-doping effect to CCTO ceramics on microstructure and dielectric properties^32^. The surface morphologies of 1–4 mol% Ag-doped CCTO ceramics sintered at temperatures from 975 to 1125 °C for 12 h were investigated. The sintering temperature was decreased by Ag dopant as a sintering aid in the CCTO ceramics. Moreover, the relative dielectric permittivity of Ag-doped CCTO ceramic was increased. Although the low sintering temperature and dielectric properties were improve by Ag-doping effect, it is different from the Ag-migration effect to CCTO ceramics. In this study, the metastable phase of CCTO ceramics can be achieved by controlling the sintering time. After the sintering process, a post-heating process at 700 °C for 1 h was employed to migrate the Ag materials into the CCTO ceramics. Therefore, we artificially controlled Ag-migration into the metastable phase region, which could generate the micro-electric path; as a result, we could increase the relative dielectric permittivity by a large margin. It is strongly suggested that developing the micro-electric path through the Ag-migration process into the metastable phase and grain boundaries can colossally increase the relative dielectric permittivity of the CCTO ceramics. Therefore for electronic device applications, the Ag-migration process into the metastable phase can be performed, introducing a micro-electric path to enhance the relative dielectric permittivity.

## Conclusions

In this research, the metastable phase of CCTO ceramics was designed and prepared with the intention of employing the Ag-migration process to develop a micro-electric path into the CCTO ceramics. We modulated the sintering time to prepare the metastable phase of CCTO ceramics. The EDS analysis revealed the metastable phase in the 2 h sintered CCTO ceramics; therefore, the post-heating process migrated Ag into the metastable phase. Also, the Ag element was detected in the grain boundary of CCTO ceramics sintered for 0.5 h and 12 h. Development of the micro-electric path through the Ag-migration process colossally increased the relative dielectric permittivity of the 2 h sintered CCTO ceramics. Impedance spectroscopy and *J*–*E* plot results support that the Ag-migration phenomenon occurs into the metastable phase and grain boundary, rather than in the more stable CCTO phase to grain region. Ag-migration into the metastable phase by the post-heating process in CCTO ceramics develops the micro-electric path to promote colossal relative dielectric permittivity.

## Materials and Methods

### Preparation of the as-sintered and Ag-migrated CaCu_3_Ti_4_O_12_ ceramics

CaCu_3_Ti_4_O_12_ powders were prepared by employing Ca(OH)_2_ (Aldrich, 95% purity), CuO (High Purity Chemicals, 99.99% purity), and TiO_2_ (Aldrich, 99.9% purity from rutile) powders. These stoichiometric powders were ball-milled using ZrO_2_ balls with ethyl alcohol for 24 h. The dried powders were calcined at 900 °C in air for 12 h at a rate of 5 °C/min, and then slowly cooled to room temperature. The CCTO powder was pressed (1 ton) into cylindrical pellets of 12 mm diameter and 1.5 mm thickness. The pellets were sintered in an electrical furnace at 1125 °C for 0.5 h, 2 h, and 12 h. In order to measure the dielectric properties and metallic migration, the polished CCTO specimens were coated with a conducting silver paste. The Ag-coated CCTO specimens were post-heated at 700 °C in air for 1 h to achieve Ag migration.

### Measurement of the as-sintered and Ag-migrated CCTO ceramics

The EDS experimental analysis showed migrated Ag in the metastable phase and grain boundaries. The relative dielectric permittivity (ε_r_), dielectric loss (tan δ), and complex impedance of the CCTO specimens were measured by employing an Agilent 4294A precision impedance analyzer (40 Hz–110 MHz). The complex impedance (${Z}^{\ast }$) was calculated using the expression,

1 $${Z}^{\ast }=Z^{\prime} -jZ^{\prime\prime} $$

where, Z* is the complex impedance, and Z′ and Z″ are the real and imaginary parts of the complex impedance, respectively. Transmission electron microscopy (TEM; Tecnai F30 ST field-emission gun instrument) was used to observe the atomic arrangement and SAED patterns. Energy dispersive spectroscopy (JEOL 5410, UK) was performed to investigate the chemical compositions of the CCTO ceramics. The electric field dependent leakage current density (*J*–*E*) characteristic at room temperature was measured by Keithley 6517 A electrometer/high resistance meter.

## Electronic supplementary material

Capacitance measurement video

Dataset1
