# Supplementary material for: Ag-migration effects on the metastable phase in CaCu3Ti4O12 capacitors
Source: Sci Rep. 2018 Jan 23;8:1392. doi: 10.1038/s41598-018-19241-0 (PMC5780478; doi:10.1038/s41598-018-19241-0)
Supplement: Supplementary file 2 — Dataset1 [file 41598_2018_19241_MOESM2_ESM.doc]

**Ag-migration effects on the metastable phase in CaCu_3_Ti_4_O_12_ capacitors**

Ji-Won Lee^1^, Gun-Hyun Lee^1^, Dong-Jin Shin^1^, Jinhwan Kim^1^, Soon-Jong Jeong^2^ &

Jung-Hyuk Koh^1*^

Supplementary Information


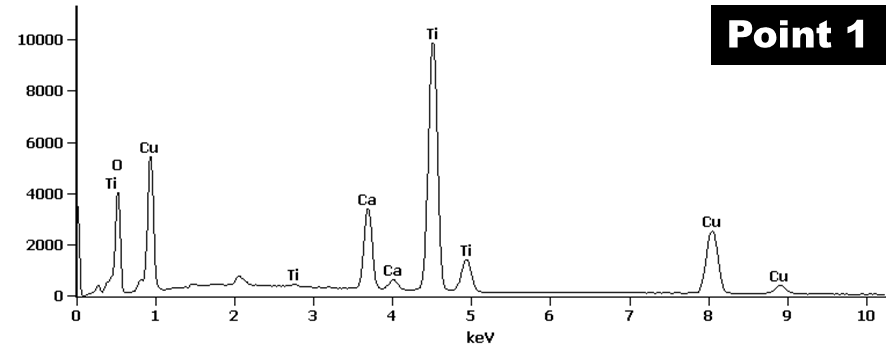


**Fig. S1**. EDS spectra of as-sintered CaCu_3_Ti_4_O_12_ ceramics sintered at 1125 °C for 0.5 h corresponding Fig. 1a.


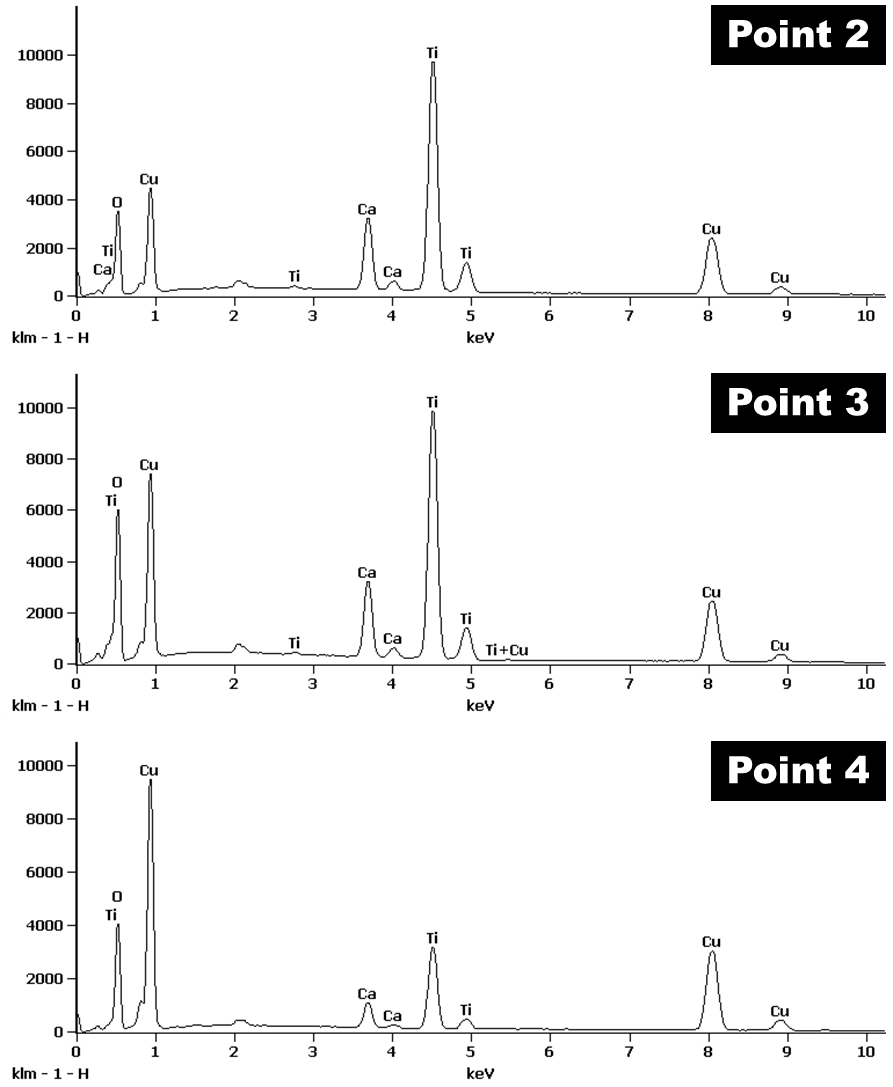


**Fig. S2**. EDS spectra of as-sintered CaCu_3_Ti_4_O_12_ ceramics sintered at 1125 °C for 2 h corresponding Fig. 1b.


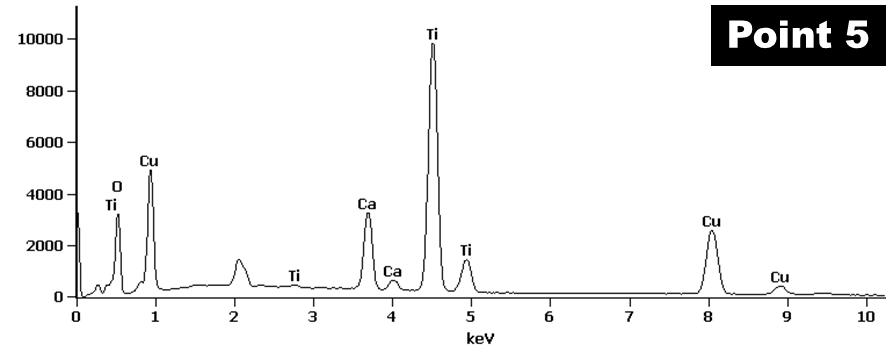


**Fig. S3**. EDS spectra of as-sintered CaCu_3_Ti_4_O_12_ ceramics sintered at 1125 °C for 12 h corresponding Fig. 1c.

**Fig. S4**. The atomic percent depending on elemental contents for grain, metastable phase and grain boundary region of CaCu_3_Ti_4_O_12_ ceramics.


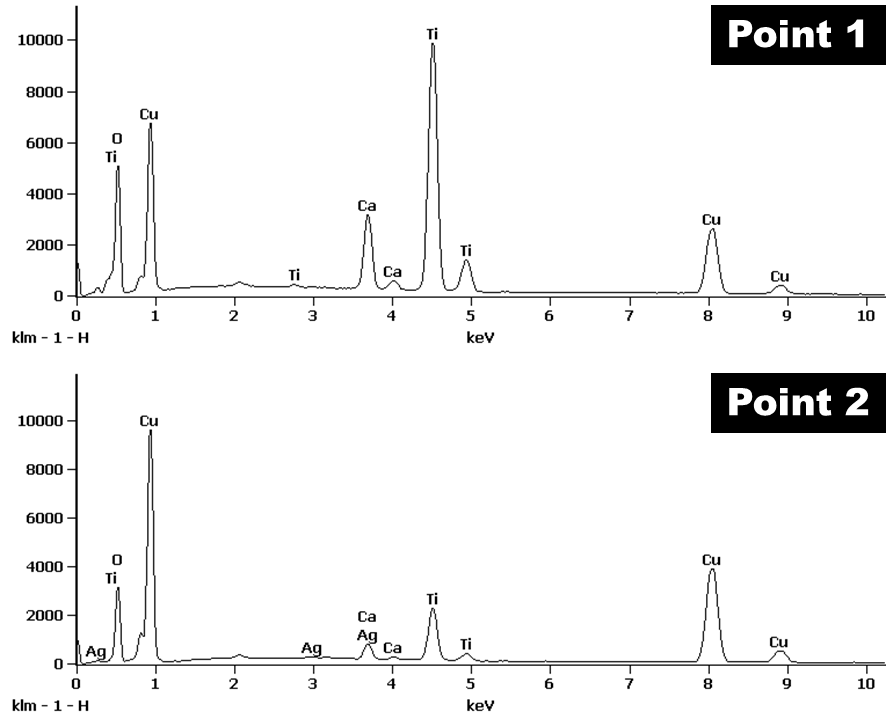


**Fig. S5**. EDS spectra of Ag-migrated CaCu_3_Ti_4_O_12_ ceramics sintered at 1125 °C for 0.5 h corresponding Fig. 3a and b.


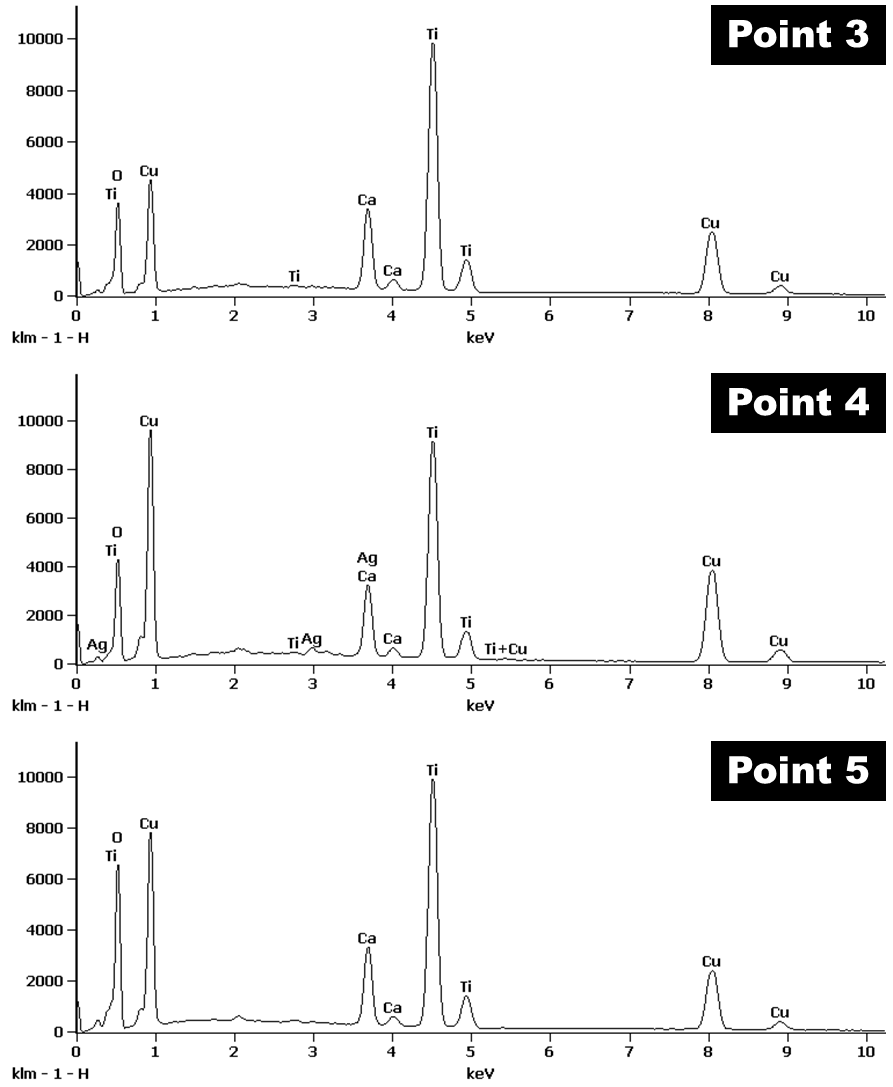


**Fig. S6**. EDS spectra of Ag-migrated CaCu_3_Ti_4_O_12_ ceramics sintered at 1125 °C for 2 h corresponding Fig. 3c and d.


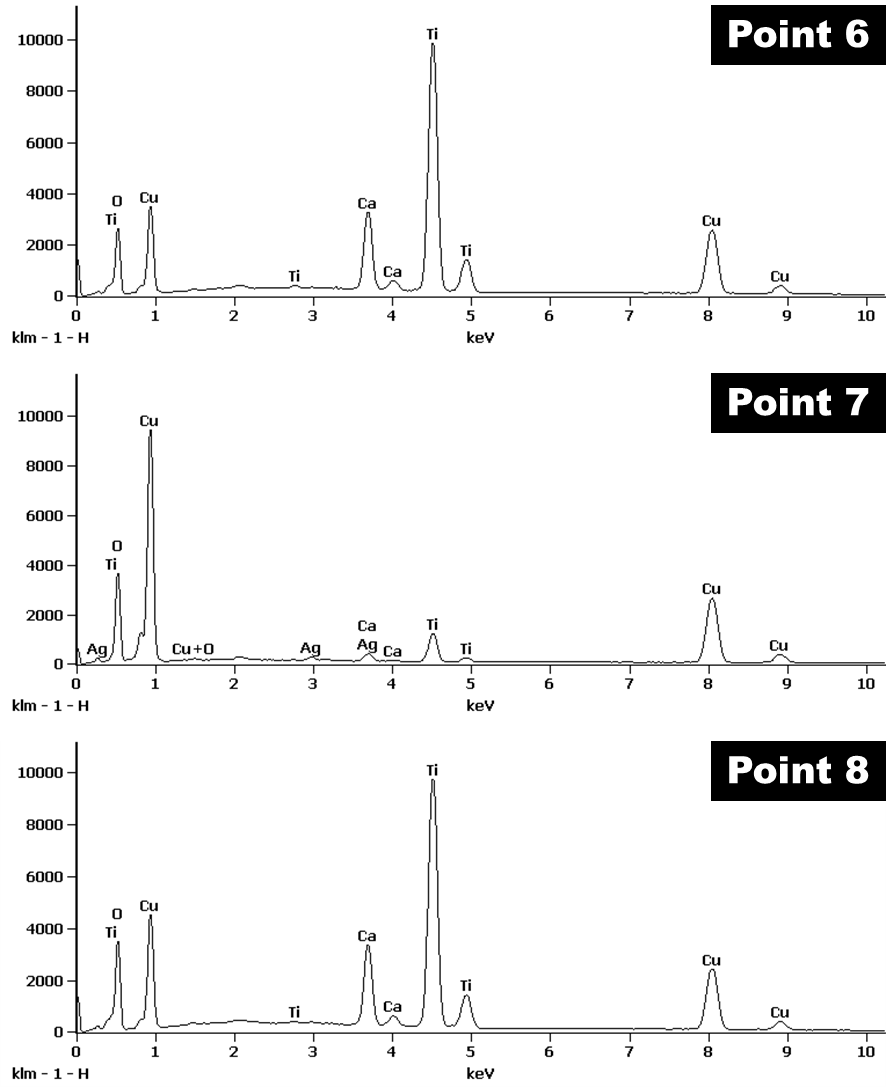


**Fig. S7**. EDS spectra of Ag-migrated CaCu_3_Ti_4_O_12_ ceramics sintered at 1125 °C for 12 h corresponding Fig. 3e and f.


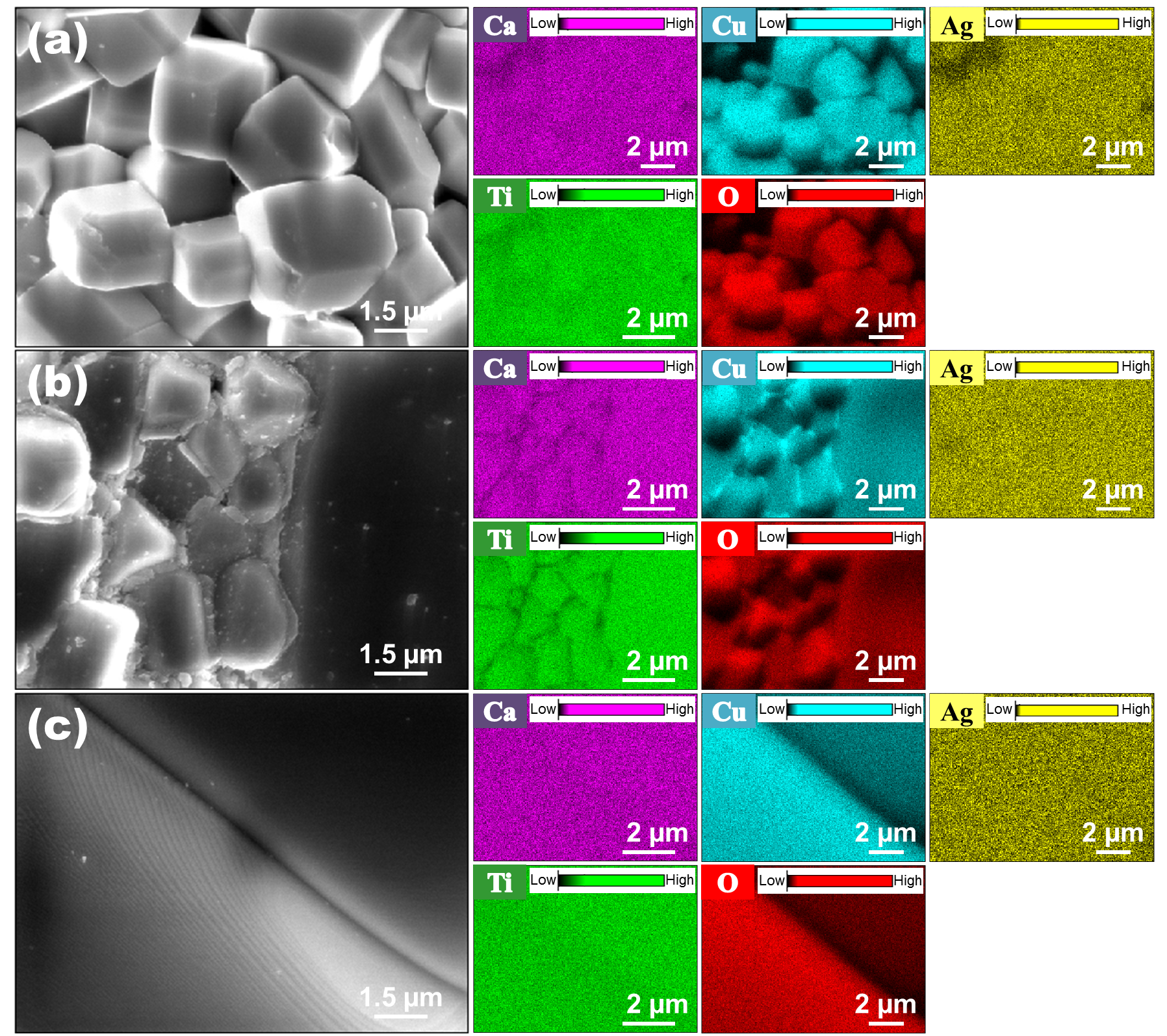


**Fig. S8**. Plane-view FE-SEM and EDS micrographs of the Ag-migrated CaCu_3_Ti_4_O_12_ ceramics sintered at 1125 °C in air for (a) 0.5 h, (b) 2 h, and (c) 12 h corresponding to the X-ray maps of calcium (Ca), copper (Cu), titanium (Ti), oxygen (O), and silver (Ag).

**Fig. S9**. Variation of imaginary part (ε") of complex dielectric constant with frequency at different sintering time of a) as-sintered and b) Ag-migrated CaCu_3_Ti_4_O_12_ ceramics.
